# Unlocking MENA’s potential in rare-disease precision medicine

**DOI:** 10.1186/s13023-026-04566-1

**Published:** 2026-09-04

**Authors:** Brahim Tabarki, Khalid Hundallah, Majid Alfadhel

**Affiliations:** 1 Division of Pediatric Neurology, Department of Pediatrics, Prince Sultan Military Medical City, Riyadh, Saudi Arabia; 2 Medical Genomic Research Department, King Abdullah International Medical Research Center (KAIMRC), King Saud Bin Abdulaziz University for Health Sciences (KSAU-HS), Ministry of National Guard Health Affairs (MNG-HA), Riyadh, Saudi Arabia; 3 Genetics and Precision Medicine Department (GPM), King Abdullah Specialized Children’s Hospital (KASCH), King Abdulaziz Medical City, Ministry of National Guard Health Affairs (MNG-HA), Riyadh, Saudi Arabia; 4 King Salman Center for Disability Research, Riyadh, 11614 Saudi Arabia

Rare diseases affect 450 million people worldwide, yet they remain understudied and underrepresented in clinical research. Although over 10,000 rare disorders are known, only a fraction have targeted therapies. Approximately 80% of these diseases have a genetic basis, whether inherited or arising de novo [1, 2]. These disorders are often chronic, posing significant challenges for affected individuals, their families, and healthcare systems. Advances in genomic technologies have greatly enhanced diagnostic capabilities, even in underrepresented regions like the Middle East and North Africa (MENA). With its unique population structure and rapidly advancing genomic capabilities, the MENA region presents a significant opportunity to transform global rare-disease precision medicine. However, despite the increasing diagnostic capabilities, international collaborative research networks remain fragmented. This fragmentation limits knowledge exchange, patient inclusion in clinical trials, and equitable access to innovation. This paper outlines the enabling factors, existing barriers, and a practical blueprint for how the MENA region can lead a new era of equitable, data-driven discovery.

## Why does MENA matter?

The MENA region holds significant potential for advancing precision medicine in rare diseases for several reasons:

### MENA region: contribution to clinical genomics

The Arab region has emerged as a critical contributor to the global discovery of rare genetic disorders [3, 4]. This is driven by its unique population structure, characterized by high rates of consanguinity, large sibships, and prominent founder effects due to strong clan affiliation. These demographic and cultural features create an enriched environment for identifying novel disease genes, delineating distinct clinical phenotypes, and discovering previously unreported pathogenic variants, including founder variants specific to certain clans or geographic regions. Research conducted in this region has expanded the catalog of human genetic variation and challenged conventional assumptions regarding modes of inheritance. For example, genes historically associated with autosomal dominant inheritance have been found to manifest as autosomal recessive disorders in Arab families, indicating context-specific genetic behavior. An example of this is the KCNMA1-related severe phenotype, characterized by myoclonic epilepsy, developmental delay, and cerebellar hypoplasia, which was identified as a homozygous inheritance pattern in the Arab population [5]. This contrasts with earlier reports from Western countries, where the same gene was thought to cause disease through dominant inheritance, presenting with a milder phenotype. Such findings emphasize the importance of considering population-specific genomic data in diagnostic interpretation, variant classification, and genetic counseling. The contribution of Arab genomic research thus extends beyond regional relevance, providing important details about genotype-phenotype correlations, allelic heterogeneity, and the broader architecture of human inherited diseases. Adding genomic data from the MENA region will also help address the prominent bias in existing variant databases, which are predominantly based on Western populations and thus limited in representing global genetic diversity. In populations with high rates of consanguinity, such as those in the MENA region, there is a greater prevalence of recessive genetic disorders, which allows for more consistent genetic backgrounds among affected individuals. This genetic homogeneity can support more precise and effective clinical trial designs and provide unique opportunities for precision medicine approaches. The inclusion of consanguineous populations in clinical research is therefore critical not only for advancing scientific understanding but also for ensuring equitable access to novel treatments for rare diseases globally.

### Arab countries: contribution to research publications

In Arab countries, medical research publications have been increasing by 87% annually from 2017 to 2022. The median impact factor of the top 20 medical journals that included Arab-affiliated authors was 5.14, and 50% of these journals were classified as quartile-one journals. When looking at the details of publications, a good proportion were related to rare diseases, with 15% in biology and biochemistry, 6% in molecular biology and genetics, and 5% in neuroscience [6].

### Collaboration of research groups and the MENA region

Despite a growing number of genetic studies emerging from the MENA region, the overall involvement of MENA-based researchers in high-impact international collaborations on rare diseases remains disproportionately low. Over the past decade, joint research initiatives between MENA and Western institutions, particularly in medical genomics and rare diseases, have increased gradually but remain modest quantitatively and thematically. For example, in developmental and epileptic encephalopathies, MENA researchers contribute to fewer than 10% of global collaborative publications. A similar trend appears in neurodevelopmental disorder research. This underrepresentation stems from systemic barriers, including restrictive data-sharing policies, data privacy issues, insufficient integration within global research networks, and, in some countries, underdeveloped genomic infrastructure. Successful models such as the International Rare Diseases Research Consortium (IRDiRC) Task Force can adapt to MENA-Western partnerships through joint training and data governance frameworks [7]. This adaptation promotes interoperational clinical research networks. Limited MENA participation may risk leaving thousands of novel variants unreported and unrecognized. This is evidenced by the fact that 34% of Arab founder variants are absent from gnomAD, thereby delaying progress in global precision medicine [8].

### Outlook: redefining global equity in genomics

The MENA region is poised to play a crucial role in global precision medicine. Its unique genetic architecture and rapidly expanding research ecosystem can accelerate discoveries not only for regional populations but also for the entire world.

Strengthening collaborative frameworks between institutions in the MENA region and global genomic and rare disease consortia is essential. This will ensure equitable representation in rare disease research, promote capacity building, and accelerate the discovery of novel disease mechanisms. To achieve this, a MENA-focused rare disease research consortium with standardized data-sharing protocols should be established, alongside funding models prioritizing low-resource regions through public-private partnerships or international grants. Ethical considerations, such as ensuring community consent for genetic studies in culturally sensitive regions, must guide these efforts. Greater integration of clinical and genomic data from institutions in the MENA region into global genomic consortia would bring significant mutual benefits. This integration would enhance the expansion of deep clinical phenotyping and genotyping, as well as the understanding of underlying pathophysiologic mechanisms for the development of specific targeted precision therapies, which would benefit all. Without concerted efforts to bridge this gap, the global genomic landscape is at risk of being skewed and incomplete. By fostering inclusive, collaborative research ecosystems, we can transform rare disease care, ensuring that no patient, regardless of geography, is left behind.

To move from regional potential to measurable clinical impact, the MENA region requires not only greater participation in global rare-disease research, but a structured implementation model. We propose the creation of a MENA Rare Disease Precision Medicine Network organized around five operational pillars: governance, harmonized clinical–genomic data, federated data-sharing, clinical implementation, and outcome measurement. Such a network should not function as a repository of regional variants, but as a learning health system linking affected families, clinicians, diagnostic laboratories, researchers, policy-makers, and patient representatives.

First, governance should be based on regional ownership and accountable international partnership. A steering committee should include clinical geneticists, disease-area specialists, bioinformaticians, ethicists, health-system leaders, and patient–family representatives from several MENA countries. This body would define membership criteria, authorship and benefit-sharing rules, data-access procedures, material-transfer agreements, and mechanisms for dispute resolution. A regional data-access committee should review requests for controlled-access data, ensuring that collaborations are scientifically justified, and ethically approved. This is particularly important in communities where family structure, tribal affiliation, consanguinity, and founder variants may increase identifiability and require culturally sensitive consent procedures.

Second, data-sharing should be designed as a federated model rather than a simple export of data to external databases. Each participating center could retain custody of identifiable clinical and genomic data while contributing standardized, queryable metadata to a shared regional infrastructure. A minimum dataset should include Human Phenotype Ontology terms, ORPHA/OMIM identifiers, inheritance pattern, pedigree structure, variant description using HGVS nomenclature, ACMG/AMP classification, zygosity, segregation data, available functional evidence, and clinical actionability. Tiered access would allow public sharing of aggregate variant frequencies and de-identified summary data, controlled access to case-level clinical-genomic datasets, and highly protected access to sensitive pedigree-linked or recontactable data. Alignment with international interoperability frameworks such as IRDiRC recommendations, GA4GH standards, ClinVar/ClinGen submission pathways, and clinical data exchange would allow MENA data to become globally useful without compromising regional sovereignty.

Third, implementation should proceed through a staged pathway. In phase 1, participating countries should map existing rare-disease clinics, diagnostic laboratories, registries, sequencing capacity, biobanks, and ethics-review processes. This phase should produce common consent templates, standardized phenotyping forms, laboratory quality-control criteria, and a regional variant-curation board. In phase 2, the network should integrate genomic testing into priority clinical pathways, beginning with high-burden specialties such as pediatric neurology, inherited metabolic disease, neurodevelopmental disorders, cardiomyopathies, immunodeficiencies, and syndromic congenital anomalies. Multidisciplinary genomic boards could review unsolved cases, reclassify variants of uncertain significance, prioritize functional studies, and identify families eligible for international matchmaking or clinical trials. In phase 3, mature centers should support therapy-oriented activities, including genotype-based trial referral, natural-history cohorts, newborn or early-childhood diagnostic pilots for actionable disorders, cascade testing, and reproductive counseling where appropriate and ethically acceptable.

Fourth, the network should be evaluated using explicit health-system indicators. Relevant metrics include the time from first specialist visit to molecular diagnosis, diagnostic yield by disease group, proportion of variants reclassified over time, number of novel disease genes or founder variants submitted to public databases, proportion of families receiving genetic counseling, number of patients matched to clinical trials or targeted therapies, reduction in repeated diagnostic investigations, and the cost per diagnosis. These indicators would shift the discussion from symbolic representation to measurable patient benefit.

Finally, the MENA model should be positioned within, but not subordinated to, the global precision-medicine landscape. Genomics England demonstrates how national genomic infrastructure can connect rare-disease diagnosis with routine healthcare and research, while the All of Us Research Program illustrates the value of diversity-oriented population-scale data platforms and secure researcher access systems. However, the MENA opportunity is distinct: its value lies in family-based rare-disease discovery, enriched recessive inheritance, founder effects, deep phenotyping, and the possibility of linking genomic discovery directly to counseling, earlier diagnosis, and therapy access in populations that remain underrepresented in global datasets. The goal, therefore, is not only to “add MENA data” to existing resources, but to build a reciprocal precision-medicine ecosystem in which regional patients benefit clinically even as global variant interpretation becomes more accurate and equitable.

## Data Availability

All data will be available upon reasonable request at the corresponding author.
